# Active Intermediates in Copper Nitrite Reductase Reactions Probed by a Cryotrapping‐Electron Paramagnetic Resonance Approach

**DOI:** 10.1002/anie.202005052

**Published:** 2020-06-04

**Authors:** Tobias M. Hedison, Muralidharan Shanmugam, Derren J. Heyes, Ruth Edge, Nigel S. Scrutton

**Affiliations:** ^1^ Manchester Institute of Biotechnology and School of Chemistry University of Manchester Princess Street Manchester M1 7DN UK; ^2^ BBSRC and EPSRC funded Future Biomanfacturing Research Hub Manchester Institute of Biotechnology and School of Chemistry University of Manchester Princess Street Manchester M1 7DN UK; ^3^ Dalton Cumbrian Facility The University of Manchester Cumbria UK

**Keywords:** copper center, copper nitrite reductase, electron paramagnetic resonance, metalloenzymes, redox enzyme

## Abstract

Redox active metalloenzymes catalyse a range of biochemical processes essential for life. However, due to their complex reaction mechanisms, and often, their poor optical signals, detailed mechanistic understandings of them are limited. Here, we develop a cryoreduction approach coupled to electron paramagnetic resonance measurements to study electron transfer between the copper centers in the copper nitrite reductase (CuNiR) family of enzymes. Unlike alternative methods used to study electron transfer reactions, the cryoreduction approach presented here allows observation of the redox state of both metal centers, a direct read‐out of electron transfer, determines the presence of the substrate/product in the active site and shows the importance of protein motion in inter‐copper electron transfer catalyzed by CuNiRs. Cryoreduction‐EPR is broadly applicable for the study of electron transfer in other redox enzymes and paves the way to explore transient states in multiple redox‐center containing proteins (homo and hetero metal ions).

## Graphical abstract

**Metalloenzyme catalysis**: Through the use of cryoreduction and annealing, a method of studying electron transfer in redox active metalloenzymes is developed. Combined with electron paramagnetic resonance spectroscopy, active states in the catalytic cycle of the copper containing nitrite reductases are probed.

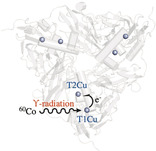

## Introduction

Transition metals (e.g. Cu, Mn, Fe and Mo) are ubiquitous in biology and play key roles in redox enzymes that are vital for life and the next generation of biofuels.^1^ Due to their complex reaction mechanism, and often, poor optical signals (e.g. the weak absorbance bands from the type‐II copper centers^2^), identifying and studying active intermediates in the reaction cycle of metal containing enzymes is challenging. Methods must be established for deeper mechanistic insights into these enzymes. Here, we develop the use of cryoreduction (with annealing at higher temperatures) in combination with electron paramagnetic resonance (EPR) spectroscopy for monitoring and detecting active intermediates in the electron transfer reactions of complex metalloenzymes.

Cryoreduction‐EPR involves the use of ionizing radiation to reduce frozen solutions of protein (e.g. 77 K). Then, by annealing the frozen protein solution to higher temperatures, reaction intermediates are formed and trapped, enabling the capture of transient enzyme species, which if paramagnetic, can be investigated using EPR spectroscopy. Active catalytic intermediates in a number of enzymes containing heme catalytic centers^3^, ^4^, ^5^ have been identified using similar cryoreduction‐EPR methods (e.g. in identification of ferric‐hydroperoxo, ferric‐peroxo, ferrous‐superoxo radical and compound I in the P450 monooxygenase reaction cycle).^3^ DNA radicals have also been investigated using cryoreduction‐EPR methods.^6^, ^7^ In this study, as a proof‐of‐principle, cryoreduction‐EPR is used to detect substrate binding, product release and the active intermediates when electrons are transferred from the type‐I to the type‐II copper centers in the copper‐containing nitrite reductase (CuNiR) family of enzymes.

CuNiRs catalyze the reduction of soluble nitrite to produce gaseous nitric oxide, a key step in denitrification and the global nitrogen cycle:^8^

(1) $$\mathrm{NO}{}_{2}{}^{-}\;+\;2\;H{}^{+}\;+\;1\;e{}^{-}\;\to \;\mathrm{NO}\;+\;H{}_{2}O$$

Two‐domain CuNiRs were first identified over 40 years ago, and have been the subject of extensive biochemical and biophysical studies.^8^, ^9^ In the last decade, a number of partner protein‐tethered CuNiRs (three‐domain CuNiRs) have been isolated and characterized.^10^, ^11^, ^12^, ^13^ These three‐domain CuNiRs maintain similar structures to their two‐domain counterparts, but contain cytochrome *c* or azurin partner proteins fused at their C‐ or N‐termini.^12^, ^13^

**Figure 1:**
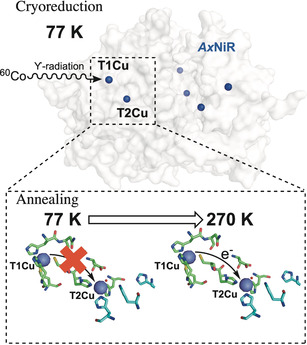
Schematic of cryoreduction‐EPR used to study inter‐copper electron transfer in CuNiRs. γ‐irradiation at a low temperature (77 K) from a ^60^Co source is used to selectively reduce the T1Cu site in CuNiRs (e.g. *Ax*NiR; PDB ID: 1OE1), and by incrementally raising and holding the temperature, electrons transfer from the T1 to the T2Cu centers. All EPR spectra were recorded at 20 K.

Two‐domain CuNiRs (e.g. the well‐characterized *Alcaligenes xylosoxidans* CuNiR; *Ax*NiR) and three‐domain cytochrome *c*‐fused CuNiRs (e.g. *Ralstonia pickettii* CuNiRs; *Rp*NiR) are trimeric enzymes.^12^, ^14^, ^15^ Within each of the three monomeric chains of CuNiRs are two β‐sandwich motifs, which both house an individual copper ion, a type I (T1Cu) and type II (T2Cu) copper. In CuNiRs, two histidine residues, a cysteine and an axial methionine residue coordinate the T1Cu center (Figure 1).^12^, ^14^ Three histidine residues and either a water (H_2_O), nitrite (NO_2_^−^) or nitric oxide (NO) molecule coordinate the T2Cu (Figure 1). Mechanistic studies show that the T1Cu receives electrons from partner proteins (in two‐domain CuNiRs) or the fused cytochrome *c*/azurin domain (in three‐domain CuNiRs).^12^, ^13^, ^16^ Electrons then transfer from the reduced T1Cu to the catalytic T2Cu ion in the core CuNiR portion of the protein, where nitrite is converted to nitric oxide.^17^, ^18^, ^19^ Inter‐copper electron transfer in CuNiRs occurs via a proton coupled electron transfer (PCET) reaction.^17^, ^18^, ^20^ Numerous methods, including laser‐flash photolysis,^17^ pulsed‐radiolysis,^18^ single‐molecule FRET,^21^ pH‐perturbation,^20^, ^22^, ^23^ and serial crystallography^24^ have been used to study inter‐copper electron transfer catalyzed by CuNiRs. Whilst these approaches have provided valuable contributions to our understanding of electron transfer in CuNiRs, they have been restricted by limited optical signal from the T2Cu center.

Here, we used a ^60^Co source of ϒ‐radiation at 77 K to cryolytically reduce the copper centers in two copper‐containing nitrite reductases, the two‐domain *Ax*NiR and the core portion of the three‐domain *Rp*NiR, in the presence and absence of the substrate, nitrite (Figure 1). Using annealing and EPR spectroscopy, simultaneous tracking of T1 and T2Cu redox centers was used to follow and probe inter‐copper electron transfer. Initially, we performed studies on “nitrite‐free” forms of the well‐characterized two‐domain *Ax*NiR and the *Rp*NiR core protein for which an X‐ray crystal structure and solution properties have been reported.^15^

## Results and Discussion

**Figure 2:**
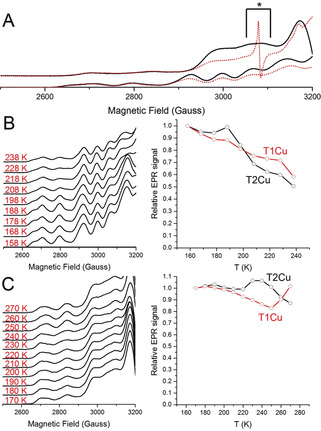
T1 to T2Cu electron transfer monitored in “nitrite‐free” *Ax*NiR and *Rp*NiR core proteins through the cryoreduction‐EPR method. A) EPR spectra of oxidized (black, solid lines) and cryolytically reduced (red, dotted lines) *Ax*NiR (bottom) and *Rp*NiR core (top) proteins. Observed EPR spectra when B) *Ax*NiR and C) *Rp*NiR core samples were annealed (left) and changes in EPR intensities of the T1 and T2Cu sites relative to the starting signal at 158 K for *Ax*NiR and 170 K for *Rp*NiR core proteins, respectively (right). All EPR spectra were recorded at 20 K. During the irradiation process, many paramagnetic EPR signals are produced and the signal indicated by the black asterisk mark in the *Rp*NiR core sample is due to the [H] radical and is formed in all the samples examined here, including both the buffer control and empty EPR quartz tubes (Figure S6).^28^, ^29^

In Figure 2 A, the EPR spectra of the oxidized “nitrite‐free” *Ax*NiR and *Rp*NiR core proteins measured at 20 K are shown. These spectra display the presence of the overlapping, four‐line, parallel hyperfine features, arising from both the T1Cu and T2Cu centers. From experimental and simulated EPR spectra (see Figure S1) collected on the *Rp*NiR core protein, it is observed that the EPR signals at the perpendicular orientation is split into four hyperfine lines, a feature that is due to the strong hyperfine coupling of the ^63, 65^Cu nuclei with the electron spin of T1Cu center. This splitting is absent in the “nitrite free” *Ax*NiR sample, suggesting subtle differences in the electronic structures of the T1Cu centers present in both of these CuNiR proteins (Figure S1 in the Supporting Information).^25^ After irradiation of frozen *Ax*NiR and *Rp*NiR proteins at 77 K with ϒ‐rays from a ^60^Co source, a reduction in the T1Cu EPR signal is observed (approx. 30 % and 50 % for *Ax*NiR and the *Rp*NiR core protein, respectively; Figure 2 and Figure S2,S3). There is a partial reduction in the EPR signal of the T2Cu site (under the conditions used here approx. 10 %; Figure 2 and Figure S2,S3), a result that is in‐line with that observed in an *in crystallo Ax*NiR study.^26^ As previously suggested, the selective reduction of the T1 over the T2Cu center is likely due to the solvent accessibility of the T1Cu, which is situated 7 Å from the surface of the protein.^17^ The T2Cu of CuNiRs is buried within the trimeric core of the protein.

In Figure 2 B, temperature‐dependent changes in the oxidized T1 and T2Cu EPR signals (measured at 20 K) for substrate‐free *Ax*NiR are presented. When *Ax*NiR samples were annealed, the intensity of the signals attributed to the Cu^II^ state of the T1Cu and the T2Cu lessened (Figure 2 B and Figure S2). Moreover, at temperatures lower than 200 K, only a small fraction of the electrons are transferred from the T1Cu to the T2Cu (<5 % of the total T2Cu signal). The majority of T2Cu reduction occurs only above 200 K, a temperature that is generally regarded as the protein “glass transition” temperature.^27^ We demonstrated recently that “solvent‐slaved” protein motions assist *Ax*NiR‐catalyzed PCET reactions.^20^ As the majority of protein motions are “frozen” below the “glass transition” temperature,^27^ the EPR data presented here provide further support for a role of protein dynamics in *Ax*NiR inter‐copper electron transfer.

Solvated electrons generated from the ϒ‐radiation in principle could directly reduce the T1 and the T2Cu centers of *Ax*NiR during the annealing process. Should this be the case, the annealing process would not report directly on T1 to T2Cu electron transfer. We therefore performed cryoreduction‐EPR measurements on a form of CuNiR that cannot transfer electrons between the copper centers in the absence of nitrite. Although the *Rp*NiR core protein is structurally similar to the *Ax*NiR protein (Figure S4), it has recently been reported that it is unable to transfer electrons between the copper centers in the absence of bound nitrite.^15^ This is attributed to the high T1Cu mid‐point potential relative to the T2Cu in the substrate free form.^15^ As such, the T2Cu of the *Rp*NiR core protein should in theory remain oxidized during the annealing process.

Temperature‐dependent changes in the T1 and T2Cu signals of the cryolytically reduced *Rp*NiR core samples are shown in Figure 2 C. Unlike the *Ax*NiR protein, there are few or no alterations in the intensity of the T2Cu signal of *Rp*NiR core during temperature ramping (between 170 and 250 K; Figure S3), indicating that solvated electrons produced from ϒ‐radiation do not directly reduce the T2Cu of the CuNiR proteins during the annealing. We do however observe a change in the electronic properties of the T2Cu center during annealing, with a second paramagnetic T2Cu species (T2Cu[2]) forming over the experimental temperature range (Figure S5). Between 170–250 K, the signal attributed to the T1Cu continuously decreases until it plateaus at a minimum. Unexpectedly, between 250 and 270 K, we observe what we hypothesize to be electron transfer from the fully reduced T1Cu to the T2Cu[2] species, causing a shift in the T2Cu equilibrium position towards the “resting” T2Cu species (T2Cu[1]) accompanied by a decrease in the T2Cu hyperfine intensity (Figure S5).

In our earlier studies, we reported that T1 to T2Cu electron transfer was inhibited in the *Rp*NiR core protein in absence of nitrite.^15^ This hypothesis was based on recorded mid‐point potentials and a laser flash photolysis assay, which was used to monitor changes in the UV/Vis active T1Cu site when laser pulses were used to rapidly inject the protein with electrons. Using the EPR approach presented here to probe the T2Cu site, we have been able to observe an additional previously uncharacterized T2Cu species, T2Cu[2], present in the *Rp*NiR core protein, which is formed upon reduction of the T1Cu and can facilitate inter‐copper electron transfer. We must note that in our cryoreduction‐EPR experiments performed on the *Rp*NiR core protein, the percentage signal change attributed to the reduction of the T1Cu site during annealing is far lower than that seen in the *Ax*NiR sample (approx. 15 % in *Rp*NiR core and 40 % in the *Ax*NiR sample). We attribute this to a larger percentage of T1Cu reduced in the *Rp*NiR core protein during the initial cryolytic reduction process (approx. 30 % and 50 % for *Ax*NiR and the *Rp*NiR core protein, respectively; Figure 2 and Figure S2,S3), a result that is due to different amounts of exposed ^60^Co‐ϒ irradiation on the samples (22 kGy in *Ax*NiR and 50 kGy in *Rp*NiR core), and also plausibly, a result of redox potential differences for the T1Cu centers in the different constructs (+255 mV in *Ax*NiR,^30^ and +331 mV in the *Rp*NiR core^15^).

**Figure 3:**
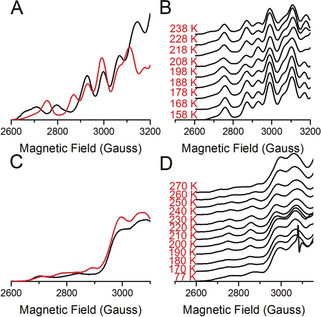
T1 to T2Cu electron transfer monitored in “nitrite‐bound” *Ax*NiR and *Rp*NiR core proteins through the cryoreduction‐EPR method. A) EPR spectra of oxidized “nitrite‐free” (black, solid line) and “nitrite‐bound” (red, solid line) *Ax*NiR. B) EPR spectral changes showing T1 to T2Cu electron transfer when “nitrite‐bound” *Ax*NiR samples were annealed. C) EPR spectra of oxidized “nitrite‐free” (black, solid line) and “nitrite‐bound” (red, solid line) *Rp*NiR core. D) EPR spectral changes showing T1 to T2Cu electron transfer when “nitrite‐bound” *Rp*NiR samples were annealed. All EPR measurements were performed at 20 K.

We also performed cryolytic reduction‐EPR measurements on “nitrite‐bound” forms of *Ax*NiR and the *Rp*NiR core proteins. Continuous wave EPR spectra of “nitrite‐bound” *Ax*NiR and the *Rp*NiR core proteins are shown in Figures 3 A and C, respectively. As expected, in the *Ax*NiR protein sample containing nitrite, the T2Cu, and not the T1Cu center is altered (A_∥_(^63,65^Cu; T2); ≈370 MHz with *g*=2.290; *Ax*NiR). This is indicative of the nitrite being bound to the catalytic T2Cu site. For the “nitrite‐bound” *Rp*NiR core protein, there are subtle changes in the T2Cu hyperfine features. Based on our new EPR spectral simulations (Figure S7), approx. 20 % of the T2Cu hyperfine features have shifted from a “nitrite‐free” to a “nitrite‐bound” state when the oxidized *Rp*NiR core was incubated with 5 mm nitrite. No additional changes in the hyperfine features of the T2Cu center were observed upon the addition of supplementary nitrite, suggesting that in an oxidized state only a fraction of the *Rp*NiR core population can accept nitrite. Previous studies have shown that a conserved tyrosine residue, present on a cyt *c* linking region, blocks nitrite from binding to the T2Cu site of the oxidized full‐length *Rp*NiR protein.^15^ This tyrosine residue is present in the *Rp*NiR core protein, but occupies an alternative state in the X‐ray structure, allowing nitrite to bind.^15^ We hypothesize that in solution, this linker and tyrosine residue may occupy multiple conformations, both blocking and allowing access of the nitrite substrate to the T2Cu center.

Temperature dependent changes in the oxidized T1 and T2Cu EPR signals (Cu^II^) of “nitrite‐bound”, cryolytically reduced *Ax*NiR are presented in Figure 3 B, Figure S2 (absolute EPR signal) and S8 (relative EPR signal). Like the “nitrite‐free” samples, and indicative of the involvement of protein motions in inter‐copper electron transfer of CuNiRs, there is little or no electron transfer below the “glass‐transition” temperature (Figure 3 B, S2 and S8). However, when samples are annealed to 198 K and then further to 218 K, there is an increase in the number of electrons transferred from the T1Cu to the T2Cu centers (shown by an approx. 30 % reduction in the T2Cu signal and an increase in the intensity of the T1Cu signal, Figure S2 and S8). There is no change in EPR line width, and only a slight change in spectral line position of T2Cu EPR signal during this electron transfer process (Figure 3 B). Based on the spectroscopic properties of the species seen at 198 and 218 K, it is likely that it is associated with the reduction of nitrite to nitric oxide and the dissociation of the product from the active site. It has been proposed that nitrite reduction at T2Cu and release of the product, nitric oxide, involves the consumption of two protons.^17^, ^30^ This would lead to the formation of either [Cu^I^−NO^+^] or [Cu^II^−NO] redox state at the T2Cu, neither of which are EPR active. The observation of a decrease in intensity in T2Cu EPR signal with A_∥_(^63, 65^Cu; T2); ≈370 MHz with *g*=2.290 also rules out the formation of [Cu^I^−NO] redox state, which would be observed *g*≤2.00 region, as in the case of *Rhodobacter sphaeroides* NiR (*Rs*NiR),^31^ and end‐on bound Cu^I^−NO inorganic model complex,^32^ with strong hyperfine coupling to the ^14^N nucleus of paramagnetic NO center; [*A*_x_(^14^N)=46 MHz; *A*_y_(^14^N)=80 MHz and *A*_z_(^14^N) not observed; *Rs*NiR & *A*_x_(^14^N)=98 MHz; *A*_y_(^14^N)=19 MHz and *A*_z_(^14^N)=17 MHz; Cu^I^−NO inorganic model complex]. Alternatively, as hypothesized previously,^17^, ^18^, ^20^ the proton coupled electron transfer to the nitrite‐bound T2Cu center leads to nitrite reduction and release of the product, nitric oxide, with the formation of [Cu^II^−OH] redox state at the T2Cu center. Between 218 and 238 K, the signal associated with oxidized nitrite‐bound T2Cu “grows in” once again (Figure 3 B, S2 and S8). We suggest that this increase in EPR signal associated with the T2Cu center is indicative of nitrite binding to the T2Cu after reduction of nitrite to nitric oxide, and subsequent nitric oxide dissociation.

Oxidized (Cu^II^) T1 and T2Cu signals of the “nitrite‐bound”, cryolytically reduced *Rp*NiR core protein are presented in Figure 3 D, Figure S3 (absolute EPR signal) and S9 (relative EPR signal). Between 150–180 K, a reduction of both the T1 and T2Cu signals is observed, a result that suggests inter‐copper electron transfer in the *Rp*NiR core protein occurs in the absence of protein motions. Above 180 K, there is an inverse trend in the signals attributed to the oxidized T1 and T2Cu centers (Figure S3 and S9). From 180–210 K, the intensity of the oxidized T1Cu decreases, while the T2Cu^II^ signal increases. However, between 210–270 K, a decrease in the oxidized T2Cu signal and an increase in the population of oxidized T1Cu are observed. Based on these data, we propose the following mechanism for the *Rp*NiR core protein. In the oxidized form of the enzyme, 20 % of the T2Cu centers are occupied with nitrite. The reducing conditions available for both T1Cu and T2Cu centers lead to the depopulation of the resting state EPR signal of the T2Cu center. This reduction of the T1Cu site appears to support binding of the nitrite substrate at the T2Cu site (shown by a shift in electronic properties and an increase in the Cu^II^ signal at the T2Cu site). Following this, electrons transfer from the T1Cu to the T2Cu site, which is now fully occupied with nitrite.

In recent work, we proposed that electron delivery to the tethered *Rp*NiR heme cofactor causes conformational change that is required for nitrite binding and catalysis in 3‐domain NiRs.^15^ We have also emphasized differences in catalytic mechanism between the full‐length and core *Rp*NiR proteins, which highlight previously unforeseen effects of tethering on enzyme catalysis. Here, we show that in the absence of the heme domain, the T1Cu of the *Rp*NiR core protein must be partially or fully reduced to enable nitrite binding and catalysis, which provides new insight into the mechanisms of 2‐domain NiRs. For example, it has been shown that values for steady‐state Michaelis constants (approx. 10 μm)^17^ during catalysis and dissociation constants (approx. 350 μm)^33^ for oxidized 2‐domain NiR–nitrite complexes differ significantly. We have used a 2‐domain NiR that (in the oxidized state) is only partially occupied with nitrite prior to performing cryoreduction‐EPR. Our studies have shown that T1Cu reduction stimulates nitrite binding to the catalytic T2Cu center. This result likely accounts for the disparity in values of the Michaelis constants and NiR–nitrate complex dissociation constants noted above.

## Conclusion

In summary, the cryoreduction‐EPR method can be used to track inter‐copper electron transfer in the copper nitrite reductase family of enzymes. Our approach highlights the importance of protein dynamics in inter‐copper electron transfer catalyzed by two‐domain copper nitrite reductases. To the best of our knowledge, this is the first spectroscopic study that has enabled direct monitoring of electron delivery, nitrite‐binding and nitric oxide production at the T2Cu in this family of enzymes. Our work has also enabled simultaneous observation of electron transfer between the T1 and T2 coppers in CuNiRs. The approach we have developed is general and could be used to further understand intra‐protein electron transfer in other multi‐center copper‐containing enzymes that have a minimal UV/Vis optical signal associated with the T2Cu sites such as laccases,^34^ peptidylglycine α‐amidating monooxygenases^35^ and particulate methane monooxygenases.^36^

## Conflict of interest

The authors declare no conflict of interest.

## Supporting information

As a service to our authors and readers, this journal provides supporting information supplied by the authors. Such materials are peer reviewed and may be re‐organized for online delivery, but are not copy‐edited or typeset. Technical support issues arising from supporting information (other than missing files) should be addressed to the authors.

Supplementary

## References

[1] K. J. Waldron , J. C. Rutherford , D. Ford , N. J. Robinson , Nature 2009, 460, 823. 19675642 10.1038/nature08300

[2] E. I. Solomon , D. E. Heppner , E. M. Johnston , J. W. Ginsbach , J. Cirera , M. Qayyum , M. T. Kieber-emmons , C. H. Kjaergaard , R. G. Hadt , L. Tian , Chem. Rev. 2014, 114, 3659–3853. 24588098 10.1021/cr400327t PMC4040215

[3] R. Davydov , T. M. Makris , V. Kofman , D. E. Werst , S. G. Sligar , B. M. Hoffman , J. Am. Chem. Soc. 2001, 123, 1403–1415. 11456714 10.1021/ja003583l

[4] S. L. Gantt, I. G. Denisov, Y. V. Grinkova, S. G. Sligar, *Biochem. Biophys. Res. Commun* **2009**, *387*, 169–173. 10.1016/j.bbrc.2009.06.154 PMC2753498 19591804

[5] R. Davydov , V. Kofman , H. Fujii , T. Yoshida , M. Ikeda-saito , B. M. Hoffman , J. Am. Chem. Soc. 2002, 124, 11120–11128. 10.1021/ja0122391 11853459

[6] A. Adhikary , D. Khanduri , M. D. Sevilla , J. Am. Chem. Soc. 2009, 131, 8614–8619. 19469533 10.1021/ja9014869 PMC2735011

[7] A. Amitava , A. Kumar , S. A. Munafo , D. Khanduri , M. D. Sevilla , Phys. Chem. Chem. Phys. 2009, 12, 5353–5368. 10.1039/b925496j PMC4677782 21491657

[8] W. G. W. Zumft , Microbiol. Mol. Biol. Rev. 1997, 61, 533–616. 9409151 10.1128/mmbr.61.4.533-616.1997 PMC232623

[9] S. S. Eady , R. R. Hasnain , Comprehensive Coordination Chemistry II, Elsevier, Amsterdam, 2003, pp. 759–786.

[10] M. J. Ellis , J. G. Grossmann , R. R. Eady , S. S. Hasnain , J. Biol. Inorg. Chem. 2007, 12, 1119–1127. 17712582 10.1007/s00775-007-0282-2

[11] S. Horrell , D. Kekilli , R. W. Strange , M. A. Hough , Metallomics 2017, 9, 1470–1482. 28702572 10.1039/c7mt00146k

[12] S. V. Antonyuk , C. Han , R. R. Eady , S. S. Hasnain , Nature 2013, 496, 123–126. 23535590 10.1038/nature11996 PMC3672994

[13] M. Nojiri , Y. Xie , T. Inoue , T. Yamamoto , H. Matsumura , K. Kataoka , Deligeer , K. Yamaguchi , Y. Kai , S. Suzuki , Proc. Natl. Acad. Sci. USA 2007, 104, 4315–4320. 17360521 10.1073/pnas.0609195104 PMC1838599

[14] S. V. Antonyuk , R. W. Strange , G. Sawers , R. R. Eady , S. S. Hasnain , Proc. Natl. Acad. Sci. USA 2005, 102, 12041–12046. 16093314 10.1073/pnas.0504207102 PMC1189323

[15] T. M. Hedison , R. T. Shenoy , A. I. Iorgu , D. J. Heyes , K. Fisher , G. S. A. Wright , S. Hay , R. R. Eady , S. V. Antonyuk , S. S. Hasnain , et al., ACS Catal. 2019, 9, 6087–6099. 32051772 10.1021/acscatal.9b01266 PMC7007197

[16] M. Nojiri , H. Koteishi , T. Nakagami , K. Kobayashi , T. Inoue , K. Yamaguchi , S. Suzuki , Nature 2009, 462, 117–120. 19890332 10.1038/nature08507

[17] S. Brenner , D. J. Heyes , S. Hay , M. A. Hough , R. R. Eady , S. S. Hasnain , N. S. Scrutton , J. Biol. Chem. 2009, 284, 25973–25983. 19586913 10.1074/jbc.M109.012245 PMC2757998

[18] S. Suzuki , Deligeer , K. Yamaguchi , K. Kataoka , K. Kobayashi , S. Tagawa , T. Kohzuma , S. Shidara , H. Iwasaki , J. Biol. Inorg. Chem. 1997, 2, 265–274.

[19] S. Suzuki , K. Kataoka , K. Yamaguchi , Acc. Chem. Res. 2000, 33, 728–735. 11041837 10.1021/ar9900257

[20] N. S. Hedison , T. M. Heyes , D. J. Shanmugam , M. Iorgu , A. I. Scrutton , Chem. Commun. 2019, 55, 5863–5866. 10.1039/c9cc01026b 31049498

[21] Ł. Krzemiński , L. Ndamba , G. W. Canters , T. J. Aartsma , S. D. Evans , L. J. C. Jeuken , J. Am. Chem. Soc. 2011, 133, 15085–15093. 21863850 10.1021/ja204891v

[22] N. G. H. Leferink , R. R. Eady , S. S. Hasnain , N. S. Scrutton , FEBS J. 2012, 279, 2174–2181. 22536809 10.1111/j.1742-4658.2012.08601.x

[23] S. Ghosh , A. Dey , Y. Sun , C. P. Scholes , E. I. Solomon , J. Am. Chem. Soc. 2009, 131, 277–288. 19053185 10.1021/ja806873e PMC2629382

[24] S. Horrell , S. V. Antonyuk , R. R. Eady , S. S. Hasnain , M. A. Hough , R. W. Strange , IUCrJ 2016, 3, 271–281. 10.1107/S205225251600823X PMC4937782 27437114

[25] B. D. Howes , Z. H. L. Abraham , D. J. Lowe , T. Brüser , R. R. Eady , B. E. Smith , Biochemistry 1994, 33, 3171–3177. 8136351 10.1021/bi00177a005

[26] M. A. Hough , S. V. Antonyuk , R. W. Strange , R. R. Eady , S. S. Hasnain , J. Mol. Biol. 2008, 378, 353–361. 18353369 10.1016/j.jmb.2008.01.097

[27] G. A. Ringe , D. Petsko , Biophys. Chem. 2003, 105, 667–680. 14499926 10.1016/s0301-4622(03)00096-6

[28] R. Davydov , K. J. Labby , S. E. Chobot , D. A. Lukoyanov , B. R. Crane , R. B. Silverman , B. M. Hoffman , Biochemistry 2014, 53, 6511–6519. 25251261 10.1021/bi500485z PMC4204881

[29] H. A. Schwarz , J. Chem. Educ. 1981, 58, 101–105.

[30] N. G. H. Leferink , C. Han , S. V. Antonyuk , D. J. Heyes , S. E. J. Rigby , M. A. Hough , R. R. Eady , N. S. Scrutton , S. S. Hasnain , Biochemistry 2011, 50, 4121–4131. 21469743 10.1021/bi200246f

[31] O. M. Usov , Y. Sun , V. M. Grigoryants , J. P. Shapleigh , C. P. Scholes , J. Am. Chem. Soc. 2006, 128, 13102–13111. 17017790 10.1021/ja056166n

[32] K. Fujisawa , A. Tateda , Y. Miyashita , K. I. Okamoto , F. Paulat , V. K. K. Praneeth , A. Merkle , N. Lehnert , J. Am. Chem. Soc. 2008, 130, 1205–1213. 18179210 10.1021/ja075071d

[33] Z. H. L. Abraham , B. E. Smith , B. D. Howes , D. J. Lowe , R. R. Eady , Biochem. J. 1997, 324, 511–516. 9182711 10.1042/bj3240511 PMC1218459

[34] D. M. Mate , M. Alcalde , Microb. Biotechnol. 2017, 10, 1457–1467. 27696775 10.1111/1751-7915.12422 PMC5658592

[35] S. T. Prigge , R. E. Mains , B. A. Eipper , L. M. Amzel , Cell. Mol. Life Sci. 2000, 57, 1236–1259. 11028916 10.1007/PL00000763 PMC11146793

[36] M. O. Ross , F. MacMillan , J. Wang , A. Nisthal , T. J. Lawton , B. D. Olafson , S. L. Mayo , A. C. Rosenzweig , B. M. Hoffman , Science 2019, 364, 566–570. 31073062 10.1126/science.aav2572 PMC6664434

