# Supplementary material for: Active Intermediates in Copper Nitrite Reductase Reactions Probed by a Cryotrapping‐Electron Paramagnetic Resonance Approach
Source: Angew Chem Int Ed Engl. 2020 Jun 4;59(33):13936–40. doi: 10.1002/anie.202005052 (PMC7497095; doi:10.1002/anie.202005052)
Supplement: Supplementary file 1 — Supplementary [file ANIE-59-13936-s001.pdf]

Angewandte Chemie
Eine Zeitschrift der Gesellschaft Deutscher Chemiker

Supporting Information

Active Intermediates in Copper Nitrite Reductase Reactions Probed by a Cryotrapping-Electron Paramagnetic Resonance Approach

*Tobias M. Hedison+, Muralidharan Shanmugam+, Derren J. Heyes, Ruth Edge, and Nigel S. Scrutton**

anie_202005052_sm_miscellaneous_information.pdf

EXPERIMENTAL METHODS

All reagents were of analytic grade and were purchased from Sigma-Aldrich (Dorset, UK). *Alcaligenes xylosoxidans* (*Ax*NiR) and the core region of *Ralstonia pickettii* copper containing nitrite reductase (*Rp*NiR core) were expressed and purified using previously published protocols.[1,2] All EPR samples were prepared in 50 mM potassium phosphate buffer (pH 7.0) within an anaerobic glovebox (< 5 ppm O_2). Prior to EPR measurements, protein samples were brought into the anaerobic glovebox, where they were oxidised with a few grains of potassium ferricyanide and subsequently passed down a Bio-Rad (Hertfordshire, UK) Econo-Pac 10DG-desalting column to remove surplus oxidant. Samples containing *approx.* 400 μM NiR (± 5 mM KNO_2/KCl), 50 mM N-methyl-nicotinamide (NMN) and 100 mM tert-butanol were transferred into 4 mm Suprasil quartz EPR tubes (Wilmad LabGlass) and sealed inside a glovebox, before being removed and frozen in liquid N_2. NMN has previously been used to study intercopper electron transfer in copper nitrite reductases,[1,3] and acts as a mediator, supporting the supply of electrons generated during radiolysis to the type-I copper centre. In previous investigations on copper nitrite reductases,[1] it was shown that in the presence of NMN, there is a significant increase of T1 and not T2Cu reduction. Tert-butanol is a scavenger, used to remove the oxidizing hydroxide radical, which is formed during radiolysis of water (see details below in Supplementary Results). Gamma-radiolysis was carried out using a Model 812 cobalt-60 source (FossTherapyServices, Inc., California, USA). The dose rate used in these experiments was 74.2 Gy/min for *Ax*NiR and 99.3 Gy/min for *Rp*NiR, and irradiations were undertaken in liquid nitrogen in batches of up to 5 kGy before replacing the nitrogen. Approximately, 22 kGy and 50 kGy of radiation were produced at 77 K for *Ax*NiR and *Rp*NiR core proteins, respectively. These absorbed dose rates of the samples were determined using a Radcal Corporation (Calafornia, USA) Accu-Dose+ base unit, equipped with a 10X6-0.18 ion chamber and checked using Fricke dosimetry. All EPR samples were measured on a Bruker ELEXYSYS-500/580 X-band EPR spectrometer with the microwave power set to 30 dB, the modulation amplitude set to 5 G, a time constant of 41 ms, a conversion time of 41 ms, a sweep time of 84 s,

the receiver gain set to 60 dB and an average microwave frequency of 9.384 GHz. The signal-to-noise in all our EPR measurements was exceptionally high. In our weakest sample (The *Rp*NiR core; Figure 2, 3 and S6), there was a signal-to-noise ratio of approximately 15:1 and 150:1 for the T2 and T1Cu centres, respectively. In the *Ax*NiR protein, the signal-to-noise was approximately 40:1 and 200:1 for the T2 and T1Cu centres, respectively (Figure 2 and 3). However, as the EPR signal associates with the T1 and T2Cu centres were integrated, the noise in these measurements is negligible. All annealing measurements were performed using a 1-propanol and liquid nitrogen solvent mixture, and all samples were annealed for approximately 2 minutes at the temperature stated. As a control, we measured at a specific annealing temperature for an additional 8 minutes. No noteworthy differences were observed between the sample annealed for 2 minutes and the one annealed for a further 8 minutes (Figure S10). EPR data were collected at 20 K. The analysis of the continuous wave EPR spectra and simulations were performed using EasySpin toolbox (5.2.18) for the Matlab program package.[4] We must emphasize that our approach, like the majority of other cryotrapping-EPR approaches,[5] is semiquantitative, and no firm thermodynamic parameters were derived from these measurements. Along with other publications in this field, our approach is used to identify the active intermediate states produced during the catalytic cycle of these enzymes.

SUPPLEMENTARY RESULTS

Upon radiolysis of water, there are three radicals that are initially produced[6]:

$$H_2O \xrightarrow{\text{radiolysis}} H_2O^{\bullet +} + e^- + H_2O^* \quad \text{(Eq. S1)}$$

Following this, a series of subsequent reactions occur, which generate hydrogen atom and hydroxyl radicals (Equation S2-4). The electron generated using radiolysis (Eq. S1) loses energy via excitation and ionization of other molecules and becomes solvated (Eq. S5):

$$H_2O^{\bullet +} + H_2O \longrightarrow {}^{\bullet}OH + H_3O^+ \quad \text{(Eq. S2)}$$

$$H_2O^{\bullet +} + e^- \longrightarrow H_2O \quad \text{(Eq. S3)}$$

$$H_2O^* \longrightarrow {}^{\bullet}OH + H^{\bullet} \quad \text{(Eq. S4)}$$

$$e^- \longrightarrow e^-\,(therm) \longrightarrow e^-\,(aq) \quad \text{(Eq. S5)}$$

Reaction 2 (Eq. S2) occurs in $1.6x10^{-14}$ s at room temperature, which is faster than the recombination of $H_2O^{\cdot+}$ and e^- (aq) (Eq. 3). These species can then rapidly react with each other, leading to the production of hydrogen atoms via Reaction 6 (Eq. S6) and hydrogen and hydrogen peroxide generation via Reactions 7-9 (Eq. S7-9):

$$e^-\,(aq) + H^+ \longrightarrow H^{\bullet} \quad \text{(Eq. S6)}$$

$$H^{\bullet} + H^{\bullet} \longrightarrow H_2 \quad \text{(Eq. S7)}$$

$$e^-\,(aq) + e^-\,(aq) \longrightarrow H_2 + 2OH^- \quad \text{(Eq. S8)}$$

$${}^{\bullet}OH + {}^{\bullet}OH \longrightarrow H_2O_2 \quad \text{(Eq. S9)}$$

Many of the radicals produced during radiolysis recombine to form water. Protons and neutrons that are generated neutralize one another. Thus, the ultimate products of water radiolysis in an argon or nitrogen saturated solution are shown in Equation S10. In Eq. S10, the G values (the number produced per 100 eV of absorbed energy) for each species is shown in the parentheses:

$$H_2O \xrightarrow{\text{radiolysis}} {}^{\bullet}OH\,(2.7) + H^+\,(0.55) + e^-\,(aq)\,(2.65) + H_2O_2\,(0.7) + H_2\,(0.45) + H_3O^+\,(2.7)$$

(Eq. S10)

The solvate electron and the hydrogen atom have a reductive potential of -2.87 V and -2.30 V against the standard hydrogen electrode (SHE), respectively. The hydroxyl radical is a highly oxidising species with a reductive potential of 2.65 V vs. the SHE. Therefore, in order the produce reducing species, alcohols such as glycerol, isopropanol and tert-butanol can be used to remove hydroxyl radicals. Isopropanol also effectively scavenges hydrogen atoms, whereas tert-butanol does not. Glycerol has been shown to dramatically influence the kinetics of copper nitrite

reductases.[7] Therefore, in our investigation tert-butanol was used as a hydroxyl radical scavenger. As the hydroxyl radical has no influence on the reduction or subsequent annealing of copper nitrite reductases, it is as effective as glycerol at scavenging the hydroxyl radical.

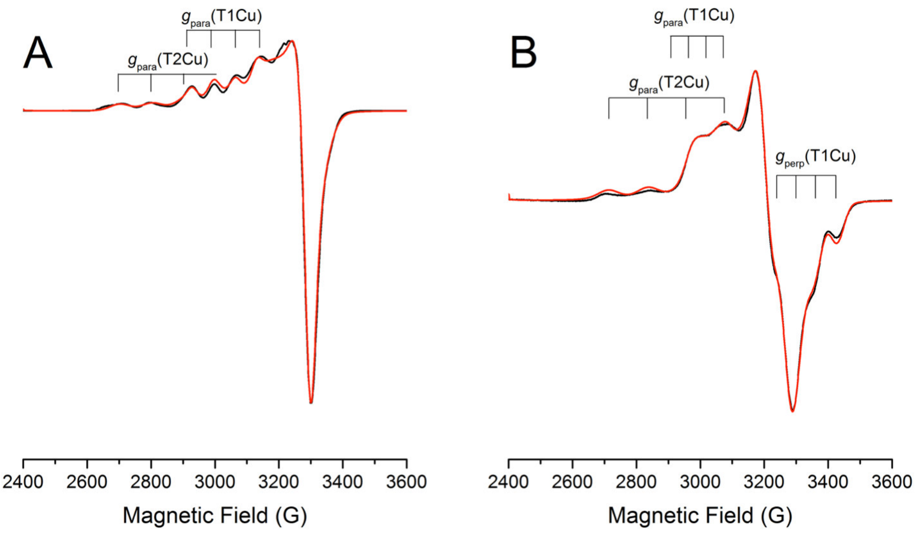

Figure S1. Experimental (black) and simulated (red) EPR spectra of the 'nitrite-free' A) *Alcaligenes xylosoxidans* and B) core region of the *Ralstonia pickettii* copper containing nitrite reductase. The 'nitrite-free' EPR spectra of both *Ax*NiR[8] and the *Rp*NiR core proteins were successfully simulated by considering two equally contributing, S = ½ spin species with the following spin-Hamiltonian parameters: T1Cu(*Ax*NiR); g = [2.036 2.049 2.212], ^{63,65}Cu(A) = [10 30 208] MHz; T2Cu(*Ax*NiR); g = [2.021 2.087 2.349], ^{63,65}Cu(A) = [29 66 330] MHz; T1Cu(*Rp*NiR-core); g = [2.017 2.060 2.214], ^{63,65}Cu(A) = [196 107 107] MHz; T2Cu(*Rp*NiR-core); g = [2.065 2.069 2.314], ^{63,65}Cu(A) = [48 50 402] MHz. As all the hyperfine features in the experimental EPR spectrum are accounted for in the simulated EPR spectrum, it is assumed that there are no free copper ions present in the buffered solutions.

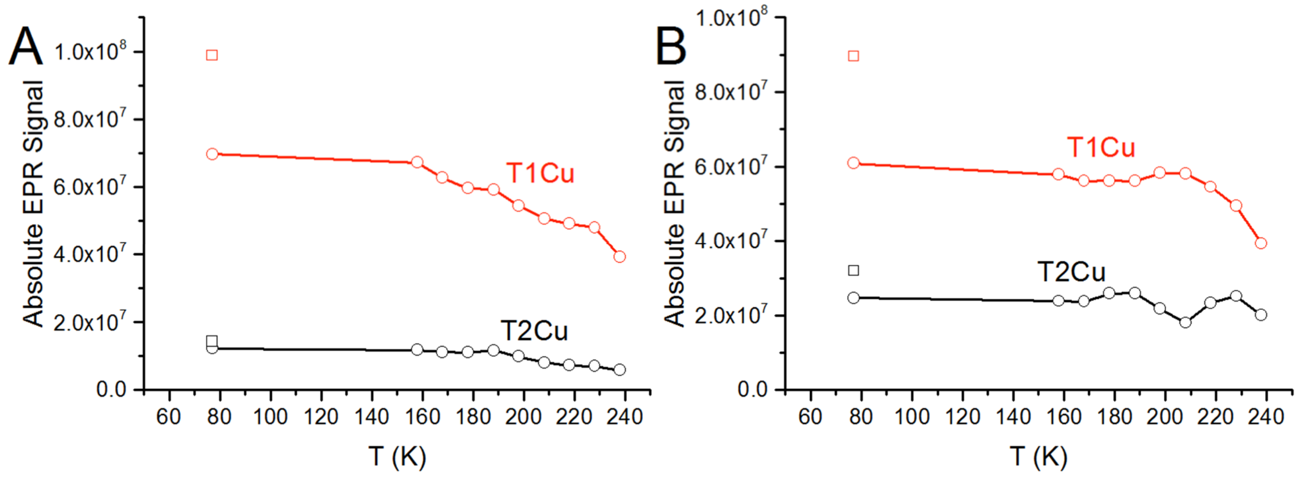

Figure S2. Temperature-dependent redox changes of the T1 and T2Cu features in A) 'nitrite-free' and B) 'nitrite-bound' *Alcaligenes xylosoxidans* copper containing nitrite reductase enzymes. Data is presented as the absolute integrated area under the T1 and T2Cu hyperfine features. The data point plotted as a square represents the intensity prior to cryo-reduction. For experimental conditions, see the experimental methods.

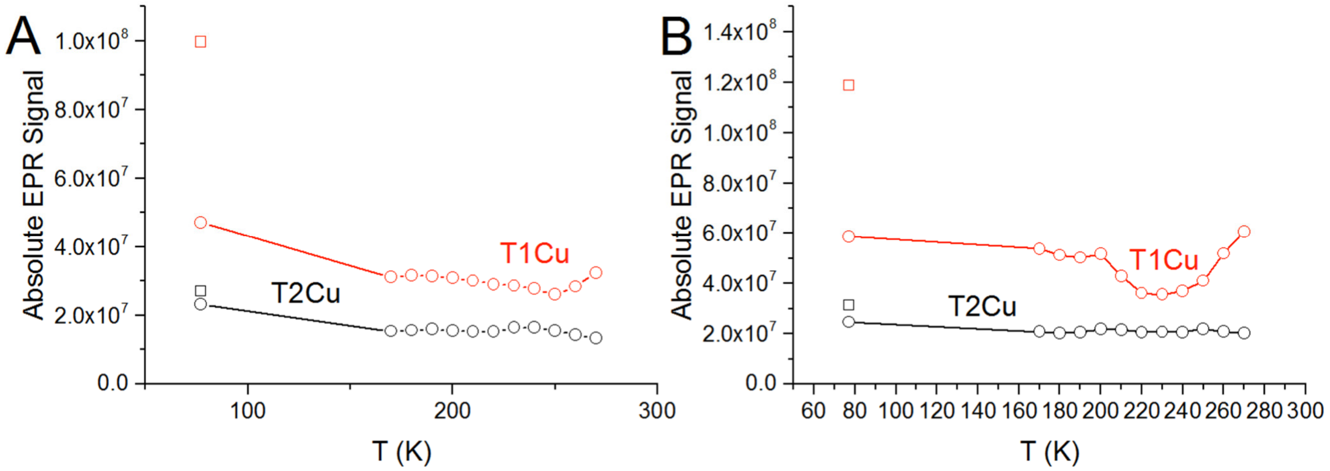

Figure S3. Temperature-dependent redox changes of the T1 and T2Cu features in A) 'nitrite-free' and B) 'nitrite-bound' *Ralstonia picketti* core copper containing nitrite reductase enzyme. Data is presented as the absolute integrated area under the T1 and T2Cu hyperfine features. The data point plotted as a square represents the intensity prior to cryo-reduction. For experimental conditions, see the experimental methods.

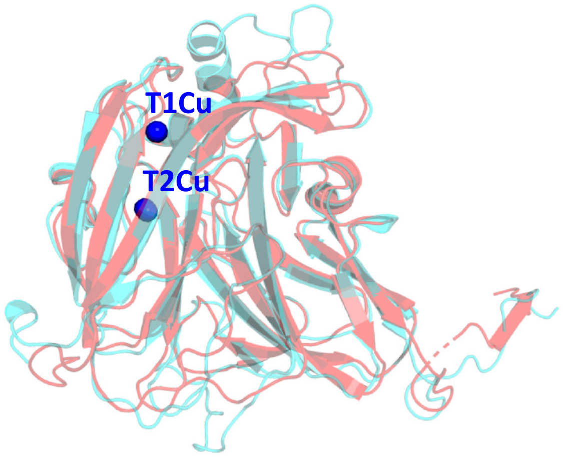

Figure S4. Structural alignment of the copper containing nitrite reductase enzymes from *Alcaligenes xylosoxidans* (blue; protein data bank ID: 1NDT) and *Ralstonia pickettii* (the core region; red; protein data bank ID: 6QPU). For simplicity, a single monomeric unit within both of the trimeric copper containing nitrite reductases is shown.

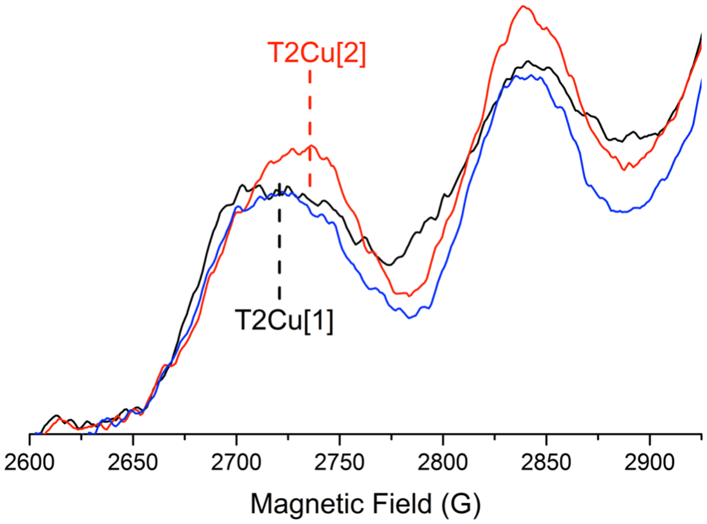

Figure S5. Formation of the T2Cu[2] species in the *Rp*NiR core protein by reduction of the T1Cu site. The black, red and blue traces correspond to the continuous wave EPR spectra recorded after annealing samples to 170, 250 and 270 K on the 'nitrite-free' *Rp*NiR core protein, respectively.

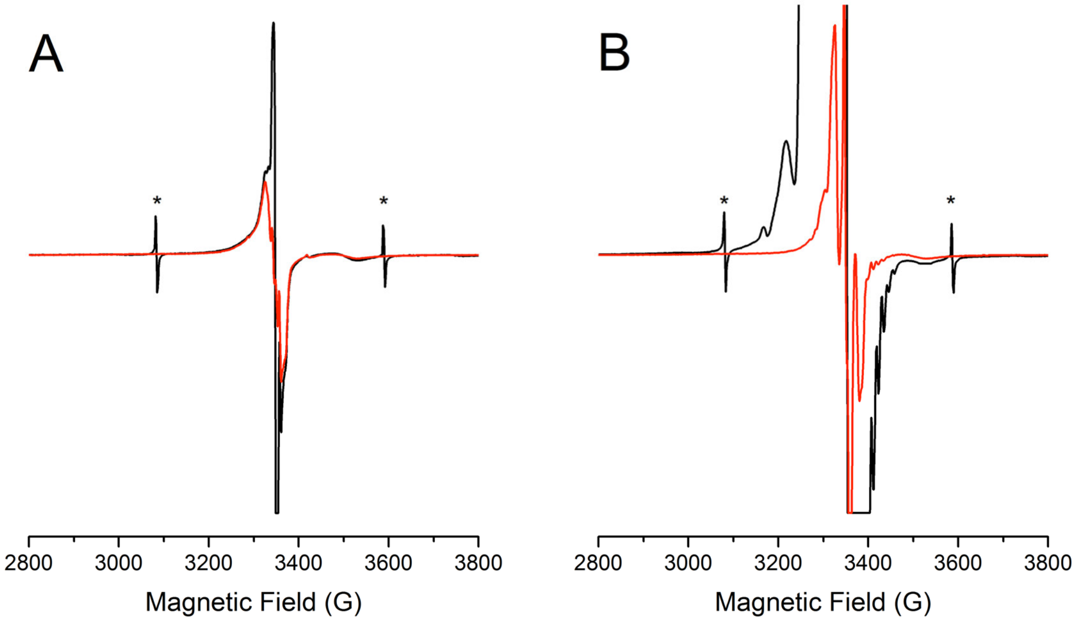

Figure S6. Continuous-wave EPR spectra displaying the paramagnetic species formed within an (A) empty EPR quartz tube, and an (B) EPR quartz tube containing 50 mM potassium phosphate buffer (pH 7.0), supplemented with 50 mM NMN and 100 mM tert-butanol when cryolytically reduced at 77 K (black) using a ^{60}Co-source and annealed to 230 K (red). The sharp doublet observed at 77 K (black traces) on both control samples, indicated by the asterisks is due to the hydrogen radical, which was completely decayed when the control samples were annealed at temperature of ≥ 200 K. The EPR data collection, ϒ-radiation and annealing experiments were performed as described in the experimental methods.

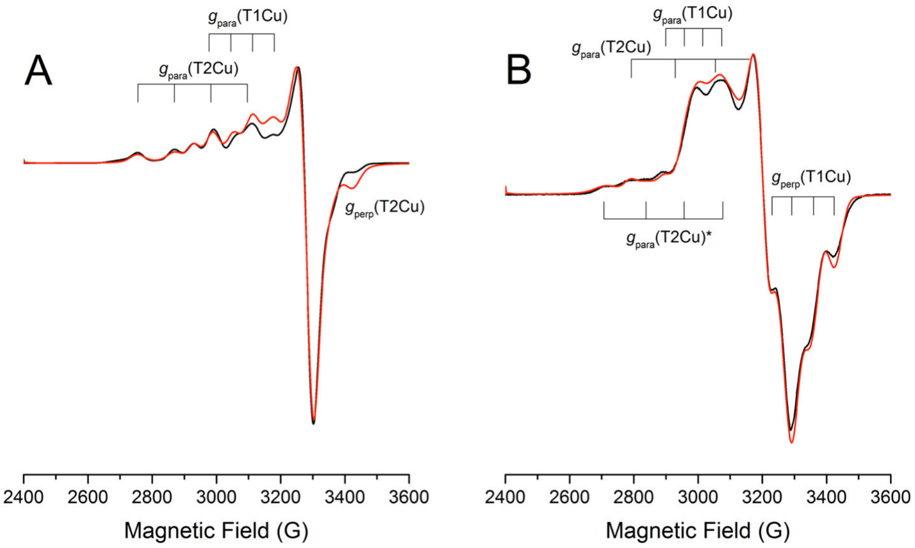

Figure S7. Experimental (black) and simulated (red) EPR spectra of the 'nitrite-bound' A) *Alcaligenes xylosoxidans* and B) core region of the *Ralstonia pickettii* copper containing nitrite reductase. The 'nitrite-bound' EPR spectrum of *Ax*NiR protein was successfully simulated by considering two equally contributing, $S = ½$ spin species with the following spin-Hamiltonian parameters: T1Cu(*Ax*NiR); g = [2.034 2.045 2.218], ^{63,65}Cu(A) = [24 19 191] MHz; T2Cu(*Ax*NiR); g = [2.023 2.045 2.290], ^{63,65}Cu(A) = [216 49 370] MHz. To simulate the 'nitrite-bound' *Rp*NiR core spectrum, additional $S = ½$ spin species is considered to account for the 'nitrite-bound' state; the parameters used and the relative weights are given below. T1Cu(*Rp*NiR core); g = [2.018 2.057 2.219], ^{63,65}Cu(A) = [196 107 107] MHz, weight = 0.635; T2Cu(*Rp*NiR core); g = [2.034 2.156 2.314], ^{63,65}Cu(A) = [48 50 402] MHz, weight = 0.269; T2Cu(*Rp*NiR core); g = [2.042 2.082 2.274], ^{63,65}Cu(A) = [10 24 340] MHz, weight = 0.177. As all the hyperfine features in the experimental EPR spectrum are accounted for in the simulated EPR spectrum, it can be assumed that there are no free copper ions present in the buffered solutions. As only approx. 20 % of the oxidised *Rp*NiR core protein can bind nitrite, the hyperfine features of the two forms of the T2Cu centre are annotated for clarity: g_{para}(T2Cu) is the 'nitrite bound' form of the T2Cu and g_{para}(T2Cu)* is the 'nitrite-free' form of the *Rp*NiR core T2Cu centre.

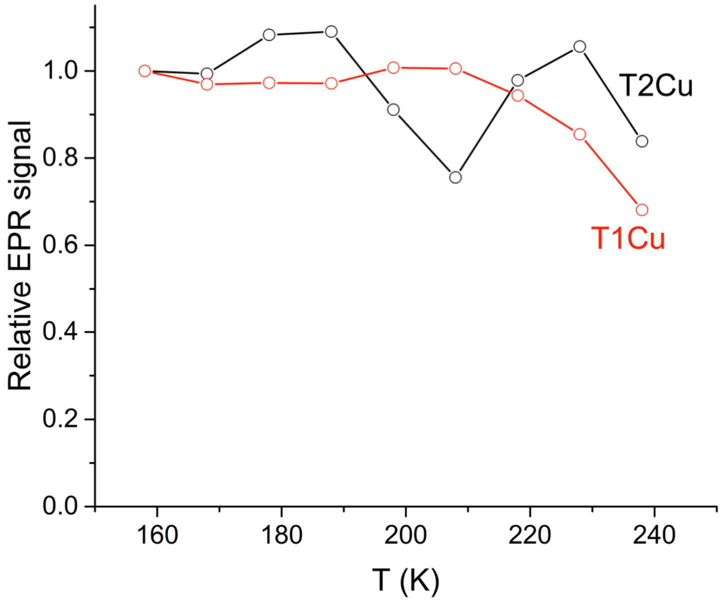

Figure S8. Temperature-dependent redox changes of the T1 and T2Cu features in the 'nitrite-bound' *Alcaligenes xylosoxidans* copper containing nitrite reductase enzymes. Data is normalised to the integrated area under the T1 and T2Cu hyperfine features at 158 K. For experimental conditions, see the experimental methods.

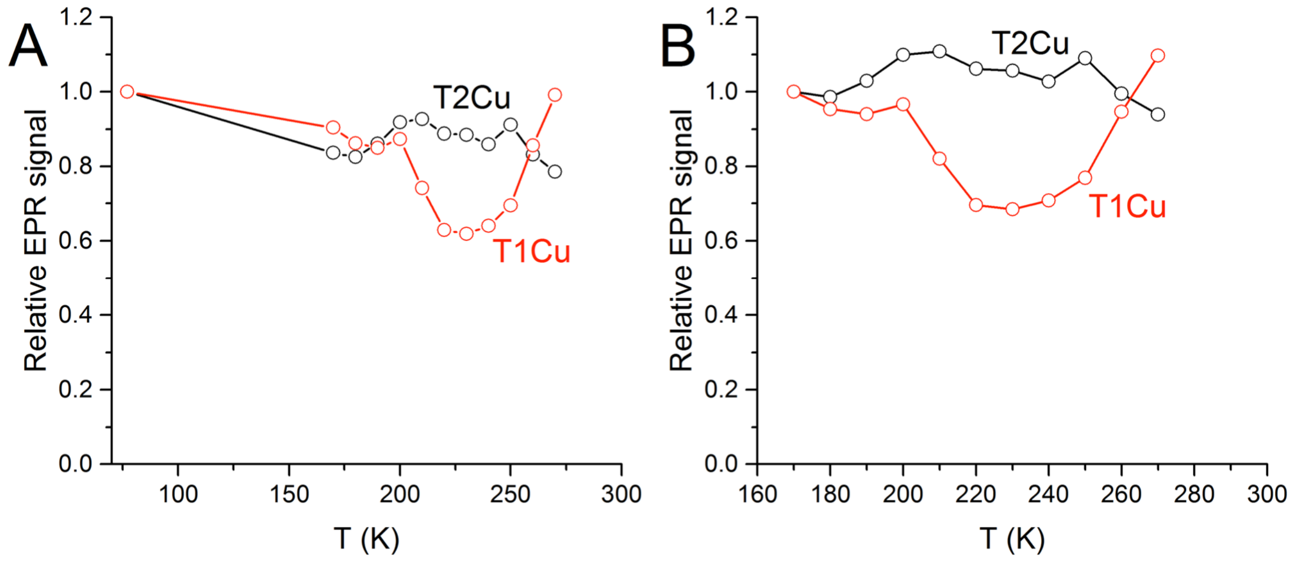

Figure S9. Temperature-dependent redox changes of the T1 and T2Cu features in the 'nitrite-bound' *Ralstonia picketti* core copper containing nitrite reducase enzyme. Data is normalised to the integrated area under the T1 and T2Cu hyperfine features at (A) 77 and (B) 170 K, respectively. For experimental conditions, see the experimental methods.

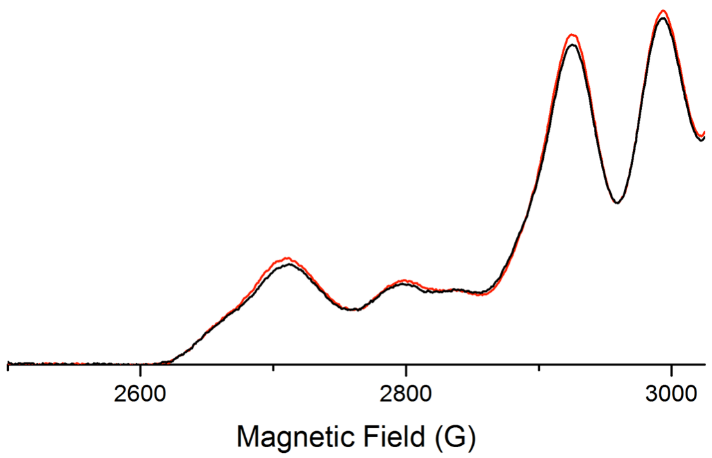

Figure S10. Example annealing measurements, showing no changes in the T1 and T2Cu hyperfine features when extending the annealing time from 2 to 10 minutes. The samples shown are for the 'nitrite-free' *Ax*NiR at 218 K. The red and black and red traces are EPR spectra recorded after 2 and 10 minutes, respectively. For experimental conditions, see the experimental methods.

AUTHOR CONTRIBUTIONS

TMH prepared all samples, designed and conceived experiments, collated, analyzed and interpreted data, wrote the manuscript, managed all experimental aspects of the project and coordinated the project. MS performed all EPR measurements and simulations, designed and conceived experiments, and analyzed and interpreted data. DH secured funding and was involved in project management. RE performed the cryoreduction experiment with MS. NSS secured funding and directed the overall project. All authors commented on the final manuscript.

REFERENCES

1 S. Brenner, D. J. Heyes, S. Hay, M. A. Hough, R. R. Eady, S. S. Hasnain and N. S. Scrutton, *J. Biol. Chem.*, 2009, **284**, 25973–25983.

2 T. M. Hedison, R. T. Shenoy, A. I. Iorgu, D. J. Heyes, K. Fisher, G. S. A. Wright, S. Hay, R. R. Eady, S. V. Antonyuk, S. S. Hasnain and N. S. Scrutton, *ACS Catal.*, 2019, **9**, 6087–6099.

3 S. Suzuki, Deligeer, K. Yamaguchi, K. Kataoka, K. Kobayashi, S. Tagawa, T. Kohzuma, S. Shidara and H. Iwasaki, *J. Biol. Inorg. Chem.*, 1997, **2**, 265–274.

4 S. Stoll and A. Schweiger, *J. Magn. Reson.*, 2006, **178**, 42–55.

5 R. Davydov, T. M. Makris, V. Kofman, D. E. Werst, S. G. Sligar and B. M. Hoffman, *J. Am. Chem. Soc.*, 2001, **123**, 1403–1415.

6 H. A. Schwartz, *J. Chem. Educ.*, 1981, **58**, 101–105.

7 N. S. Hedison, T.M.; Heyes, D.J.; Shanmugam, M.; Iorgu, A.I.; Scrutton, *Chem. Commun.*, 2019, **55**, 5863–5866.

8 B. D. Howes, Z. H. L. Abraham, D. J. Lowe, T. Brüser, R. R. Eady and B. E. Smith, *Biochemistry*, 1994, **33**, 3171–3177.
